# Laser Activated and Electroless Metalized Polyurethane Coatings Containing Copper(II) L-Tyrosine and Glass Microspheres

**DOI:** 10.3390/molecules26185571

**Published:** 2021-09-13

**Authors:** Piotr Rytlewski, Bartłomiej Jagodziński, Rafał Malinowski, Bogusław Budner, Krzysztof Moraczewski, Agnieszka Wojciechowska, Piotr Augustyn

**Affiliations:** 1 Department of Materials Engineering, Kazimierz Wielki University, 85-064 Bydgoszcz, Poland; bar.jag@ukw.edu.pl (B.J.); kmm@ukw.edu.pl (K.M.); augustyn@ukw.edu.pl (P.A.); 2 Łukasiewicz Research Network—Institute for Engineering of Polymer Materials and Dyes, 87-100 Toruń, Poland; rafal.malinowski@impib.lukasiewicz.gov.pl; 3 Institute of Optoelectronics, Military University of Technology, 00-908 Warsaw, Poland; boguslaw_budner@interia.eu; 4 Faculty of Chemistry, Wroclaw University of Science and Technology, Wybrzeże Wyspiańskiego 27, 50-370 Wroclaw, Poland; agnieszka.wojciechowska@pwr.edu.pl

**Keywords:** polymers, copper(II) L-tyrosine, glass microspheres, electroless metallization, lasers, surface activation

## Abstract

Polyurethane coatings containing copper(II) L-tyrosine and glass microspheres were laser irradiated and underwent electroless metallization. Various sizes of glass microspheres were incorporated into the polyurethane coating matrix in order to examine their effects on surface activation and electroless metallization. The surface of the coatings was activated by using ArF excimer laser emitting ultraviolet radiation (λ = 193 nm) using different number of laser pulses and their fluence. The effects of surface activation and metallization were evaluated mainly based on optical and scanning electron microcopies (SEM), energy-dispersive X-ray spectroscopy (EDX) and photoelectron spectroscopy (XPS). It was found that the presence of glass microspheres enabled the reduction in copper complex content, intensified the ablation process (higher cone-like structures created) and resulted in higher content of copper metallic seeds. On the other hand, the glass microspheres concentration, which was higher for lower size microspheres, was advantageous for obtaining a fully metallized layer.

## 1. Introduction

The metallization of non-metallic materials, especially polymers, is an important industrial process crucial in the production of modern electronic or mechatronic devices [1,2,3]. The most common metallization techniques are physical vapor deposition (PVD), chemical vapor deposition CVD or chemical metallization (electroless metallization). Among these three techniques, electroless metallization is used on the largest industrial scale [4]. These methods are essentially classified as non-selective because all surface areas are metallized. In the case of selective metallization, only dedicated areas of the surface, e.g., conductive tracks are metalized, and this process commonly requires the application of surface masking. A relatively new and widely developed technique is electroless metallization of selectively activated surface areas with the use of laser radiation.

In the conventional electroless metallization method, the surface of the dielectric material is activated non-selectively by immersion in appropriate chemical solutions. Most often, it is a mixture of tin and palladium chlorides, sometimes preceded by chemical cleaning and etching of the surface of polymeric materials [5]. This process provides the so-called surface sensitization, which enables its activation in the next wet chemical treatment. Surface activation consists in adsorption of platinum or palladium ions, most often from a salt solution of these compounds. Only such a prepared surface is ready to be metallized in a solution containing metal ions, most often copper or nickel. These techniques require several chemical baths and do not provide the surface selectivity of metallization.

For the sake of these limitations, more ecological techniques of selective metallization with the use of laser radiation are currently developed. In one variation of these methods, the surface may be laser pre-activated and then activated and chemically metallized only in the area of laser irradiation [6,7]. In another variation of these methods, the surface is directly activated by laser radiation and then directly and electrolessly metallized [8,9]. The latter group of methods requires the prior incorporation of metallization precursors into the matrix of the polymer coating or the entire volume of polymer material. This technique is often referred to molded interconnect devices (MID) manufacturing, and it has the advantage in reducing surface preparation steps, while its disadvantage refers to the need of metallization precursors which need to be co-compounded with polymer materials [10,11].

These precursors can be organic and/or inorganic compounds of metals, especially copper in the case of selective copper plating. The most commonly used inorganic compounds are copper aluminum oxide [12], copper-chromium oxide [13], copper hydroxyl phosphate [14], whereas the effectiveness of organometallic compounds has been confirmed, e.g., palladium-acetate [15], copper acetate [16] and copper acetylacetonate [17,18]. The mechanism of laser activation is, on the one hand, ablation of the matrix and organic ligands when organometallic compounds are applied, and the reduction of these metal compounds to a metallic form on the other hand. Metal agglomerates formed locally on the surface under laser radiation constitute active sites for electroless metallization.

Various types of lasers are used in laser surface activation techniques, with preference to the relatively cheap semiconductor lasers having a wavelength of 1064 nm. The range of this radiation corresponds to photons with energies of about 1.17 eV, which is significantly lower than the energy of chemical bonds in polymer materials. In that case, surface activation is purely thermal because only an increase in temperature enables the breaking of chemical bonds and ablation with the precipitation of metal agglomerates on the surface.

Currently, new directions in the development of laser activation techniques are focused on the search for new metallization precursors and on the selection of parameters for the laser irradiation process. This article meets these prospects because, on the one hand, a commercially unavailable copper complex in the form of copper(II) L-tyrosine is investigated, on the other hand, ultraviolet laser radiation is used, which is capable of breaking most chemical bonds in organic materials (λ = 193 nm/6.4 eV). These studies in this area have already been published [19,20]. However, a significantly new element presented in this study is the use of glass microspheres, which made it possible to reduce the mass fraction of the copper complex in polymer matrix. This article shows the positive effect of microspheres and presents obtained results of metallization due to their sizes.

## 2. Experimental

### 2.1. Materials

The following materials were applied in this study:

- Substrate of polymer coatings, polycarbonate (PC), trade name: Xantar 19 UR (DSM Engineering Plastics, The Netherlands), with a melt flow rate MFR = 19.4 g/10 min (1.2 kg, 300 °C) and density of 1.20 g/cm^3^;
- Matrix of polymer coatings, polyurethane resin type B4060 (Haering, Germany) with crosslinking agent B9031 (Haering, Germany);
- Additives for polymer coatings;
- Organometallic complex-copper(II) L-tyrosine ([Cu(L-tyr)_2_]_n_), self-prepared;
- Glass microspheres (Dennert Poraver GmbH, Germany);
- Six-component metallization bath, type M-Copper 85 (MacDermid-Poland, Łysomice, Poland).

### 2.2. Preparation of Test Samples

The test samples consisted of a polymer substrate and a polymer coating intended to be metallized. The polymer substrate consisted of polycarbonate plates obtained by injection molding, having a thickness of 1 mm. A TRX 80 ECO 60 injection molding machine (Tederic Inc., Hangzhou, China) was used to produce the plates, with the temperatures of zones I, II, the head and the mold set to 285, 285, 285 and 85 °C, respectively.

**Figure 1:**
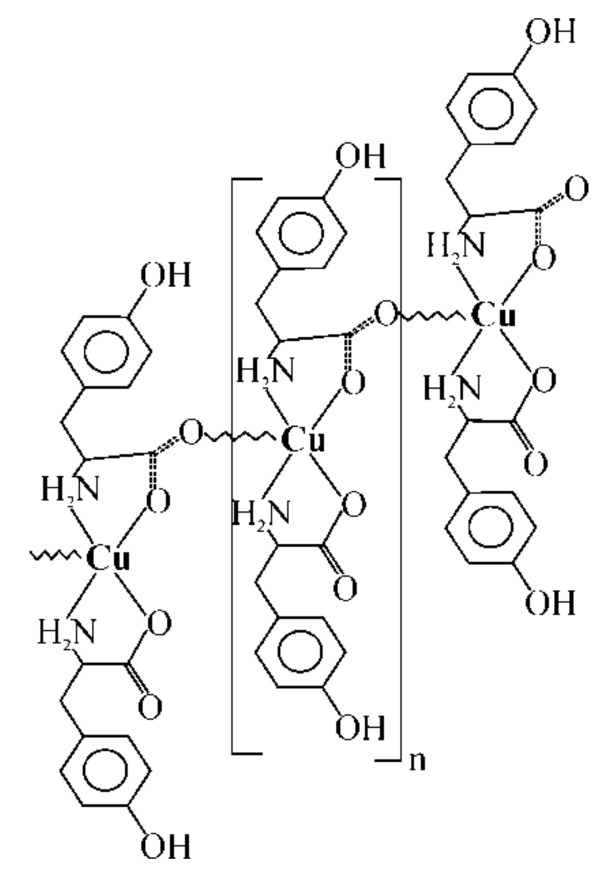
The schemes of [Cu(L-tyr)_2_]_n_.

Polymer coatings were made of liquid polyurethane resins constituting the matrix of coatings to which copper(II) L-tyrosine (15 wt.%) and glass microspheres (5 or 10 wt.%) were introduced. Copper(II) L-tyrosine was obtained by chemical synthesis, the course of which was described in earlier article [21]. The structure of the studied complex is shown in Figure 1.

The structure of the applied copper complexes was solved on the basis of the spectra determined by the KM4 X-ray diffractometer (KUMA Diffraction, UK) by numerical methods using the SHELXS-97 software. This complex was chosen because it was proved to be an effective metallization precursor, as demonstrated in recent work [19]. The obtained copper complex compound was dried at 40 °C for 24 h, crushed with a laboratory mortar, mixed with glass microspheres and polymer resin and finally evenly applied on polycarbonate plates.

Four types of glass microspheres, which differed in average size, were used for the tests. The selected physical properties of the microspheres used are listed in Table 1.

Each type of glass microspheres (Φ_1_, Φ_2_, Φ_3_ or Φ_4_) was introduced into the coatings in the amount of 5 or 10 wt.% in order to verify their effect on the reduction of copper complexes occurring under the influence of laser radiation. It also seemed interesting to determine the potential influence of the size of these microspheres on the effects of electroless metallization.

### 2.3. Laser-Induced Activation of Coatings

The coatings on the PC substrate were irradiated with an ArF LPX PRO 305 excimer laser (Coherent Inc., Santa Clara, CA, USA), with a fluence of 100 mJ/cm^2^ using 350 or 500 laser pulses. These irradiation parameters caused effective ablation and surface activation and were selected based on our previous study [20]. The applied laser fluence has a value higher than the ablation threshold energy of most polymer materials, which is about 20 mJ/cm^2^. The fluence of laser pulses was determined with a FieldMax II TOP meter (Coherent Inc., Santa Clara, CA, USA). The samples were irradiated in air under ambient conditions.

The aim of laser irradiation was to activate the surface of polymer coatings, resulting in direct electroless metallization of the irradiated areas. The origins of such activation number in two alongside physicochemical processes taking place under the influence of laser radiation: laser ablation and reduction of copper from the complex structure into the metallic one so that the precipitated copper agglomerates covers the irradiated surface of the coating, thus rendering it activated.

### 2.4. Electroless Metallization

The metallization of the irradiated samples was carried out by using a six-component electroless copper plating bath type M-Copper 85 (with formaldehyde as a reducing agent. The metallization bath was prepared in accordance with the manufacturer’s instruction, by mixing the ingredients in the correct order and quantity. The finished bath was poured into a glass vessel and heated to a temperature of about 46 °C, where the pH value was 12.8. The coatings on PC plates were metallized for 60 min. During metallization, the bath was constantly aerated.

### 2.5. Testing Techniques

Scanning electron microscopy (SEM) was performed by using the SU8010 microscope (Hitachi, Japan). In order to obtain better imaging, the tested coatings were covered with a gold layer with a thickness of about 2 nm using a resistance evaporative sputtering machine (Cressington, UK). Images of the samples were recorded at an accelerating voltage of 15 kV and a beam electron current of about 60 μA.

This microscope was also used for energy-dispersive X-ray spectroscopy (EDX). It was used to determine changes in the elemental composition of the coatings surface layer induced by laser irradiation. In order to obtain representative results of changes taking place in surface layer of the tested coatings, the surface area of 1.2 × 0.9 mm of each tested coating was scanned with an electron beam. In the case of EDX experiments, the surface of the polymer coatings was not covered with gold in order to refrain from affecting registered changes in its chemical structure.

X-ray photoelectron spectroscopy (XPS) studies were carried out by using an R3000 spectrometer (VG Scienta, Sweden). The qualitative and quantitative analysis of copper in various oxidation states was performed based on vector analysis [22,23]. This fitting technique was described in our previous studies.

Tests of the adhesive strength of coatings/deposited copper layer were carried out by tearing off the glued stamp by using the Instron 3367 testing machine (Instron, USA). Measurement stamps with dimensions of 6.5 × 20 mm and a special two-component adhesive Araldite 2011 (Huntsman, Switzerland), which is characterized by a very good ability to bond with metals and polymer materials, were used.

## 3. Results and Discussion

**Figure 2:**
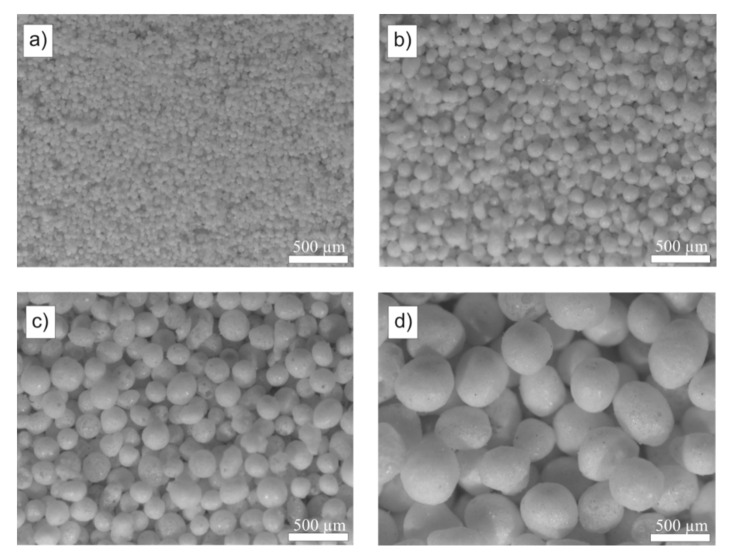
Optical images of glass microspheres: (**a**) Φ_1_, (**b**) Φ_2_, (**c**) Φ_3_ and (**d**) Φ_4_.

The sizes of glass microspheres declared by the producer (see Table 1) were verified using optical microscopy (Figure 2).

The manufacturer specifies a wide range of glass microspheres sizes. However, based on the microscopic analysis of representative groups of microspheres, it was found that their average size is close to the minimum values of the given ranges (Table 1). What is noteworthy is also a decrease in the average density of microspheres with an increase in their size, which indicates changes in the structure of the microspheres depending on their size.

**Figure 3:**
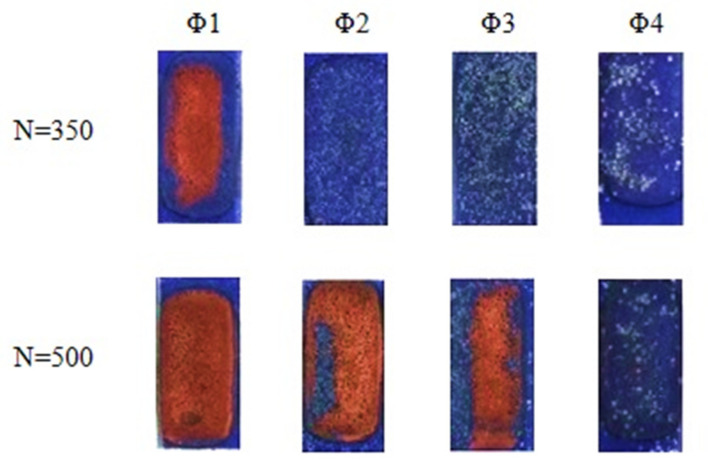
Coatings containing 15 wt.% of [Cu(L-tyr)_2_]_n_ and 5 wt.% of glass microspheres Φ_1_, Φ_2_, Φ_3_ and Φ_4_, irradiated with 350 or 500 laser pulses (N) with a fluence of 100 mJ/cm^2^, after electroless metallization.

**Figure 4:**
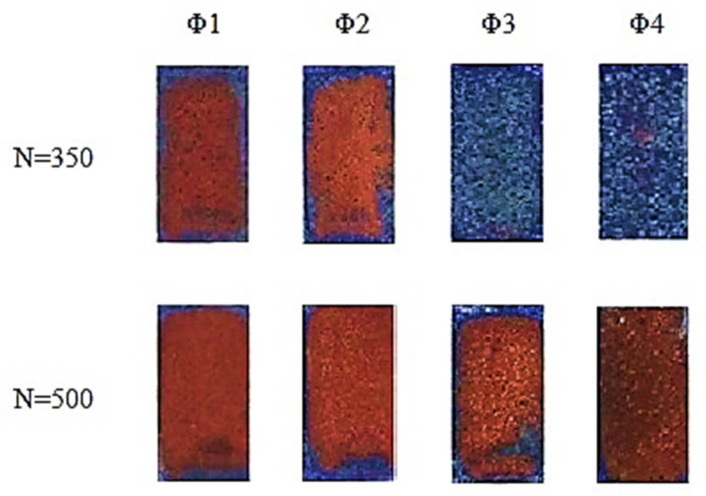
Coatings containing 15 wt.% of [Cu(L-tyr)_2_]_n_ and 10 wt.% of glass microspheres Φ_1_, Φ_2_, Φ_3_ and Φ_4_, irradiated with 350 or 500 laser pulses (N) with a fluence of 100 mJ/cm^2^, after electroless metallization.

Figure 3 and Figure 4 show the results of electroless metallization of coatings with glass microspheres with the share of 5 wt.% or 10 wt.% and with four different grain sizes.

The use of microspheres allowed the electroless deposition of copper on laser-irradiated coatings, which contained only 15 wt.%. of [Cu(L-tyr)_2_]_n_. In the case of coatings containing no glass microspheres, the content of 15 wt.% of [Cu(L-tyr)_2_]_n_ did not cause electroless metallization, regardless of the laser radiation parameters used. As presented in Figure 3 and Figure 4, the best metallization effects were obtained for coatings containing Φ_1_ glass microspheres. The larger the diameter of the glass microspheres, the less copper was deposited on the coatings as a result of electroless metallization. Better effects of electroless metallization were found in the case of coatings containing 10 wt.% of glass microspheres compared to those containing 5 wt.%.

**Figure 5:**
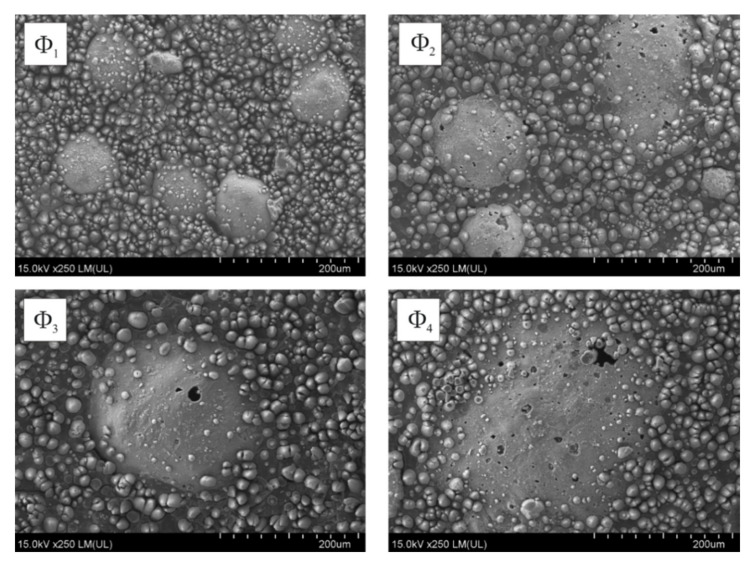
SEM images of laser irradiated (N = 500; E_j_ = 100 mJ/cm^2^) coatings containing glass microspheres: Φ_1_, Φ_2_, Φ_3_ and Φ_4_.

Coatings with 15 wt.% of [Cu(L-tyr)_2_]_n_ and 10 wt.% of glass microspheres of different sizes (Φ_1_, Φ_2_, Φ_3_ and Φ_4_) were selected for detailed analysis. These coatings were irradiated with the same number of laser pulses (N = 500) and at the same fluence (100 mJ/cm^2^) in order to determine the influence of microsphere sizes on the changes induced by laser radiation. As presented in Figure 5, laser ablation caused the uncovering of microspheres and precipitation of copper agglomerates on the coatings surface.

**Figure 6:**
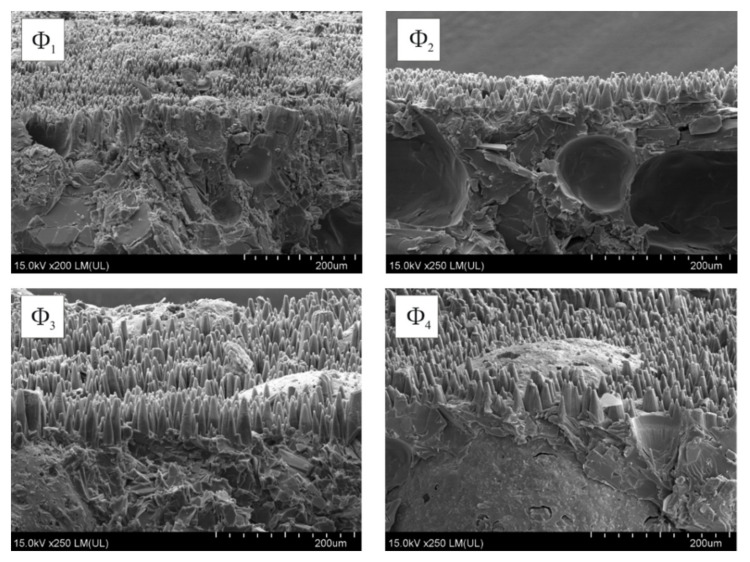
SEM images of laser irradiated (N = 500, E_j_ = 100 mJ/cm^2^) coatings containing glass microspheres: Φ_1_, Φ_2_, Φ_3_ and Φ_4_.

Under the influence of laser radiation, numerous cones were formed in the coatings surface layer. These cones are clearly visible in SEM imaging of fractured samples taking the perspective view (Figure 6).

The formation of these cones can result from the significant difference in the ablation threshold for the copper and polymer matrix. The ablation threshold for copper (2 J/cm^2^ [24]) is significantly higher than the applied laser fluence. Therefore, laser irradiation can only cause the polymer matrix to be ablated, while precipitated copper will agglomerate and stay on the surface. Along with laser pulses, copper agglomerates form a local mask on the surface, thus protecting underneath created polymer cones. Although this mechanism was explained previously, one can perceive some interesting effects of varied size of microspheres.

The height of the cones on the surface of coatings irradiated with the same laser dose increased along with the size of the glass microspheres contained in these coatings. This finding can confirm the expectation that the glass filler as highly resistant to laser ablation will result in an increase in surface temperature and, thus, to a higher ablation rate.

It can be explained by the fact that glass filler gains much higher temperatures than the polymer matrix. In the case of polymer matrix, the maximum possible temperature can be estimated at about 400 C, which refers to the thermal degradation temperature. The successive laser pulses cannot increase this value. On the other hand, the ablation threshold for glass is about 50 times higher than for polymers. Therefore, radiation energy absorbed by glass filler can be converted only into heat without mechanical effects of ablation. The higher temperature which can be gained for glass than polymer also results from the following equation [25]:

(1) $$\Delta T=\alpha \;F_{th}^{⬚}/\rho c_{⬚}\;$$

where α denotes absorption coefficient; $F_{th}^{⬚}$ denotes ablation threshold; ρ denotes material density; c denotes specific heat capacity.

With the higher temperature of glass microspheres, one can also expect higher precipitation rate of metallic copper.

**Figure 7:**
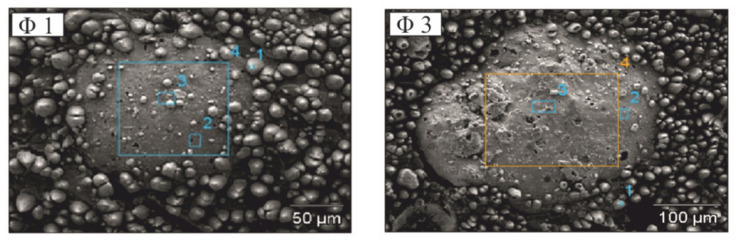
SEM images with rectangular areas on glass microspheres Φ1, Φ3 in coatings, where the amount of copper was examined by the EDX method.

Figure 7 shows the exposed areas of glass microspheres with diameters of Φ_1_ and Φ_3_ for which an EDX point analysis for the presence of copper was carried out.

The percentage of copper for the Φ_1_ microsphere area indicated in the Figure 7 was approximately 16 at%, while for the Φ_3_ microsphere it was approximately 25 at%. It is noticeable that with the increase in the size of the glass microspheres, due to irradiation with laser pulses, an increasing amount of copper is precipitated from copper L-tyrosine on the surface of the glass microspheres. This percentage of copper at the surface area of the glass microsphere itself is many times greater than the percentage of copper in the area of 1.2 per 0.9 mm of the coating, which was about 3 at%, regardless of the size of the glass microspheres used. This means that the greatest concentration of copper is on the surface of the exposed glass microspheres than between them.

**Figure 8:**
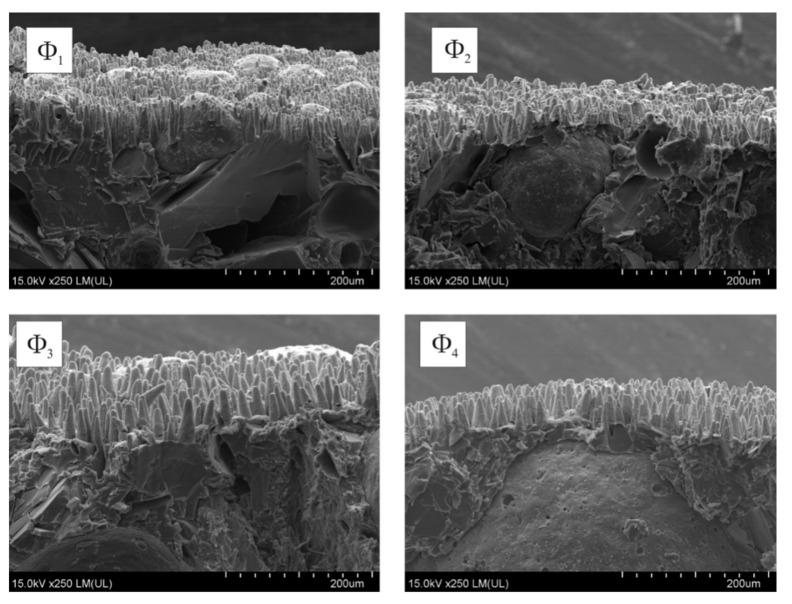
SEM images of laser irradiated (N = 500; E_j_ = 100 mJ/cm^2^) and metallized coatings containing glass microspheres: Φ_1_, Φ_2_, Φ_3_ and Φ_4_ (side view).

The deposited copper layer structure reflected the same structure as shaped upon laser irradiation (Figure 8).

The height of the cones is of about several dozen micrometres; thus, the thickness of deposited copper can be estimated to be up to a few micrometres. However, simple electrical tests showed fine and good conductivity of about 7 S/cm; thus, the deposited copper layer can be considered continuous.

Based on the XPS measurements, a slight increase in the percentage of copper atoms in the coatings containing glass microspheres of higher size was found (Table 2). With XPS spectrophotometer applied in the tests, it is not possible to analyse the only exposed areas of the glass microspheres in order to confirm the results obtained from the EDX local analysis.

As observed from Table 2, with the greater size of microspheres, the copper content slightly increased. In the analysed surface layer with a thickness of several nanometers, the share of copper in the coatings containing glass microspheres was higher than in coatings without microspheres. In coatings containing 20 wt.% (without microspheres), irradiated at the same conditions (N = 500; F = 100 mJ/cm^2^), the copper content was lower than 2 at% [20]. Therefore, higher content of copper proves that glass microspheres affected the reactions conditions on the surface. This finding is consistent with the one concerning the height of the cones, which was discussed previously when referring to Equation (1).

**Figure 9:**
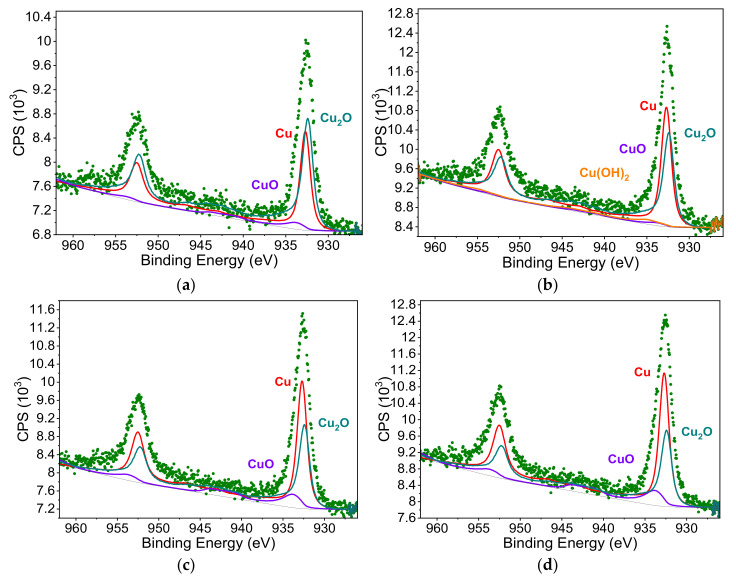
XPS spectra of laser irradiated (N = 500; Ej = 100 mJ/cm^2^) of coatings containing microspheres: (**a**) Φ_1,_ (**b**) Φ_2_, (**c**) Φ_1_ and (**d**) Φ_1_.

Figure 9 shows photoelectron spectra with matching spectra, respectively, for each of the forms of copper, Cu(0) CuO, Cu_2_O and Cu(OH)_2_, whereas in Table 3 is the percentage for each of these copper forms.

Based on the analysis of the XPS results, it was found that the copper precipitated by laser radiation occurs mainly in the form of metallic copper (Cu(0)) and in the form of copper (I) oxide (Cu_2_O). These results are significantly different from those determined for coatings containing no glass microspheres, where the share of copper in the form of Cu(0) was less than 25% and that of metallic copper was over 30% [20]. With the increasing size of the microspheres, the percentage of copper in the form of Cu and CuO increased at the expense of the Cu_2_O form in laser irradiated coatings. The differences in the chemical structure may indicate different temperature conditions in the coatings irradiated with the same dose of laser, which contained microspheres of different sizes. It is generally known that the higher the temperature, the higher the degree of copper oxidation. In the case of larger glass microspheres, ablation exposed their larger surfaces, which absorbed laser radiation, heating up to higher temperatures than the polymer matrix. Therefore, the larger the diameter of the microspheres used, the more copper is detected, both in the form of Cu(0) and CuO.

**Figure 10:**
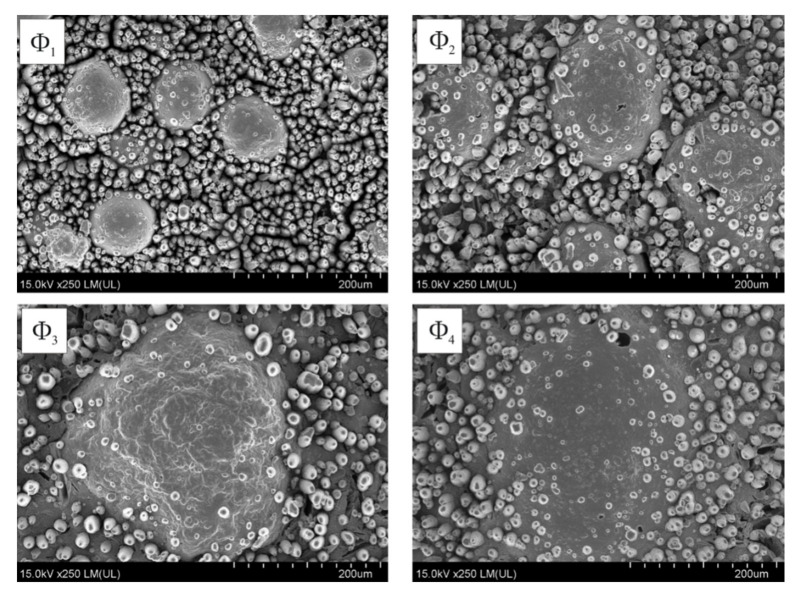
SEM images of laser irradiated (N = 500; E_th_ = 100 mJ/cm^2^) and electroless metallized coatings containing glass microspheres Φ_1_, Φ_2_, Φ_3_ or Φ_4_.

Laser irradiated coatings (N = 500, E_th_ = 100 mJ/cm^2^) underwent electroless metallization regardless of the size of the glass microspheres used. The geometric structure of the deposited copper layer reflected the geometric structure of the coating resulting from laser irradiation (Figure 10).

The EDX tests showed that the surface layer of the metallized coatings contained about 70 at% of copper, whereas the remaining proportion of oxygen and carbon atoms was in a comparable ratio of 1:2, regardless of the type of microspheres used. The EDX results for metallized coatings, however, showed significant deviation, depending on the area of the coating that was scanned. Significant local variations in the determined composition increased with the diameter of the microspheres, the size of which, especially in the case of Φ4, constituted a significant share of the scanned area.

Tests of adhesive properties were also carried out on the basis of which it was found that the adhesive strength of electroless deposited copper and polymer coating is greater than that of the polymer coating and polycarbonate substrate, because the glued stamps were detached with coatings from the PC substrate with registered adhesion strength of about 3 MPa. One can conclude that the adhesion strength of copper layer to polymer coatings has to exceeds this value.

## 4. Conclusions

The main aim of this study was to evaluate the effects of glass microspheres on laser-induced surface activation and electroless metallization. First of all, the presence of glass microspheres enabled reducing the content of copper(II) L-tyrosine, as a metallization precursor, to 15 wt.%. The size of microspheres used at the same mass content was also of importance. As proved by SEM analysis, the heights of the cones formed under laser irradiation were larger for coatings containing larger size of microspheres. Moreover, more metallic copper was precipitated on surface of larger glass microspheres. However, the final metallization effect with copper was also dependent on the surface concentration of uncovered microspheres; thus, the lower the size of microspheres, the higher their concentration. Higher concentrations of uncovered microspheres resulted in the formation of continuous metallized copper layer. For that reason, the application of lower size microspheres is more preferable, especially when higher special resolution of metallized surface area is required.

## Figures and Tables

**Table 1:** Selected physical properties of the microspheres used.

Designation	Size (µm)	Average Bulk Density (kg/m^3^)	Average Particle Density (kg/m^3^)	Crush Resistance (N/mm^2^)
Φ_1_	od 40 do 60	530+/−70	1400+/−300	-
Φ_2_	od 100 do 150	400+/−60	950+/−150	2.8
Φ_3_	od 250 do 350	340+/−30	700+/−80	2.6
Φ_4_	od 500 do 700	270+/−30	500+/−80	2.0

**Table 2:** Percentage (P) of copper, oxygen and carbon with an indication of the binding energy (E) of photoelectrons derived from these elements.

Microasphers Size	Cu		O		C	
	P (at%)	E_B_ (eV)	P (at%)	E_B_ (eV)	P (at%)	E_B_ (eV)
Φ_1_	2.7	932.66	18.8	532.56	78.5	284.96
Φ_2_	3.2	932.62	20.8	532.37	76.1	284.87
Φ_3_	3.6	932.62	19,7	532.62	76.7	285.22
Φ_4_	3.7	932.56	19.4	532.51	76.9	285.12

**Table 3:** Relative percentage (P) of individual forms of copper on the surface of coatings with glass microspheres after irradiation with laser pulses.

Coatings with Microspheres:	Cu		CuO		Cu_2_O		Cu(OH)_2_	
	P (at%)	E_B_ (eV)	P (at%)	E_B_ (eV)	P (at%)	E_B_ (eV)	P (at%)	E_B_ (eV)
Φ_1_	38.1	932.70	6.0	933.90	55.9	932.40	---	---
Φ_2_	45.1	932.70	6.7	933.90	46.2	932.40	2.0	934.95
Φ_3_	47.6	932.70	11.7	933.90	40.7	932.40	---	---
Φ_4_	49.8	932.70	13.2	933.90	37.0	932.40	---	---

## Data Availability

Data is contained within the article.
